# Optimal Design of an Hourglass in-Fiber Air Fabry-Perot Microcavity—Towards Spectral Characteristics and Strain Sensing Technology

**DOI:** 10.3390/s17061282

**Published:** 2017-06-04

**Authors:** Qi Wang, Dongchao Yan, Binbin Cui, Zixuan Guo

**Affiliations:** 1 College of Information Science and Engineering, Northeastern University, Shenyang 110819, China; 20144258@stu.neu.edu.cn (D.Y.); 20143994@stu.neu.edu.cn (B.C.); 20143761@stu.neu.edu.cn (Z.G.); 2 State Key Laboratory of Synthetical Automation for Process Industries (Northeastern University), Shenyang 110819, China; wangqi@ise.neu.edu.cn

**Keywords:** fiber Fabry-Perot interferometer, in-fiber air microcavity, spectral characteristics, strain, high fringe contrast, high resolution

## Abstract

An hourglass in-fiber air microcavity Fabry-Perot interferometer is proposed in this paper, and its second reflecting surface of in-fiber microcavity is designed to be a concave reflector with the best curvature radius in order to improve the spectral characteristics. Experimental results proved that the extinction ratio of Fabry-Perot interferometer with cavity length of 60 μm and concave reflector radius of 60 μm is higher than for a rectangular Fabry-Perot interferometer with cavity length of 60 μm (14 dB: 11 dB). Theory and numerical simulation results show that the strain sensitivity of sensor can be improved by reducing the microcavity wall thickness and microcavity diameter, and when the in-fiber microcavity length is 40 μm, the microcavity wall thickness is 10 μm, the microcavity diameter is 20 μm, and the curvature radius of reflective surface II is 50 μm, the interference fringe contrast of is greater than 0.97, an Axial-pull sensitivity of 20.46 nm/N and resolution of 1 mN can be achieved in the range of 0–1 N axial tension. The results show that the performance of hourglass in-fiber microcavity interferometer is far superior to that of the traditional Fabry-Perot interferometer.

## 1. Introduction

In recent years, a variety of fiber optic strain sensors have been studied [1,2,3] for application in biological systemx [4], structural health monitoring in composite materials [5,6] and civil engineering applications, such as health monitoring of buildings and dams [7,8]. For fiber Bragg grating (FBG) sensors, the strain sensitivity is less than 1.2 pm/με [9,10], and for fiber Mach-Zehnder interferometers, the sensitivity is about 5.0 pm/με [11,12]. However, in these sensors, the cross-sensitivity between strain and temperature is hard to overcome.

Optical microcavity sensing structures, as an alternative type of strain sensor, have unique advantages, such as high sensitivity, compact size, and low temperature cross-sensitivity [13,14,15,16]. Steinmetz et al. studied the microcavity concave mirror, which is made with miniature spherical mirrors positioned on the end of single- or multimode optical fibers by a transfer technique [17]. A concave mirror with CO_2_ laser was made in [18,19,20]. A short cavity Fabry-Perot sensor for strain sensing was also fabricated through acid etching the end of multi-mode fiber [21]. In 2007, Rao et al. studied micro-Fabry-Perot interferometers in silica fibers machined by femtosecond laser. A 75 μm cavity length based on the PCF was made through femtosecond laser and the strain sensitivity reaches 0.006 nm/με [22]. In 2012, Duan et al. took advantage of an optical fiber fusion splicer to obtain a 100 μm ellipsoid microcavity, and used it in tensile sensing, where the sensitivity was 4 pm/με and linearity was 99.99% [23]. A Fabry-Pérot (FP) strain sensor made by splicing a section of hollow-core ring photonic crystal fiber between two standard single mode fibers was investigated. For a length of 13 μm a sensitivity of 15.4 pm/με and temperature sensitivity of ~0.81 pm/°C was attained [24]. In 2013, a microhole was also fabricated in the end face of single mode fiber by femtosecond laser. Then the fiber tip with the microhole structure was spliced together with another cleaved single mode fiber. The SMF with a hollow sphere was tapered by controlling the moving speed of the flame and the holders. A maximum sensitivity of 6.8 pm/με was achieved with taper region length of 860 μm [25]. In 2014, Kaur et al. presented a microcavity strain sensor for high temperature applications. The EFPI sensor is fabricated by micromachining a cavity on the tip of a standard single-mode fiber with a femtosecond laser and is then self-enclosed by fusion splicing another piece of single-mode fiber. The sensor exhibits linear performance for a range up to 3700 με and a low temperature sensitivity of only 0.59 pm/°C through 800 °C [26].

In this work, an hourglass in-fiber air microcavity Fabry-Perot interferometer is proposed. The second reflecting surface of in-fiber microcavity is designed to be a concave reflector with the best curvature radius in order to improve the spectral characteristics. Compared with the fabrication processes and strain sensitivity of other microcavity devices [18,19,20,21,22,23,25], this sensor is attractive for its low cost, small volume, high sensitivity and better performance than the traditional Fabry-Perot interferometer. The experimental results proved that the extinction ratio of a Fabry-Perot interferometer with a microcavity length of 60 μm and concave reflector radius of 60 μm is 14 dB, and the extinction ratio of rectangular Fabry-Perot interferometer with microcavity length of 60 μm is only 11 dB. Linearity is up to 99.947% in the range of 0-1 N axial tension, and axial-pull sensitivity is up to 20.46 nm/N, the maximum interference intensity of reflection spectrum is above 0.08. The contrast of reflection spectrum is greater than 0.97, and the cavity length is 40 μm, which guarantees a good free spectral range (28 nm).

## 2. Sensor Structure and Sensing Principle

### 2.1. Sensor Structure

**Figure 1:**
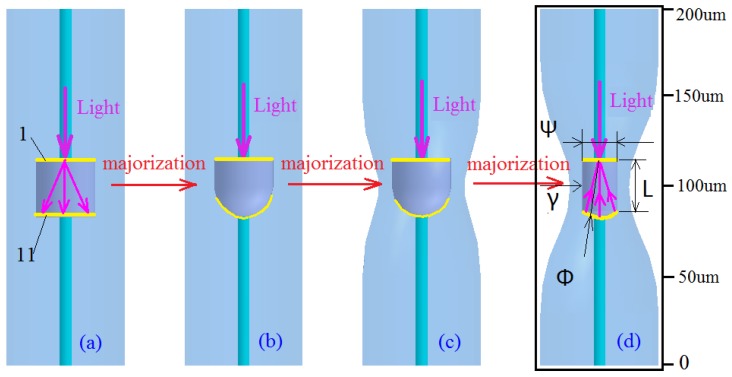
Majorization design of optical microcavity strain sensor. (SMF: the diameter of core is 8.2 μm, the diameter of cladding is 125 μm; Reflective surface I; Reflective surface II; Light: 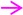) (**a**) F-P cavity with rectangular reflector; (**b**) F-P cavity with concave mirror; (**c**,**d**) Hourglass in-fiber air Fabry-Perot Microcavity.

The traditional in-fiber Fabry-Perot cavity is an axisymmetric cylinder-structure with a fiber core. The two reflective surfaces are parallel to the axial-vertical plane, and the internal material of the microcavity is air, as shown in Figure 1a. In this paper, a new kind of hourglass fiber air microcavity structure is designed in single-mode fiber (Corning SMF-28e+), the structure is shown in Figure 1d.

**Figure 2:**
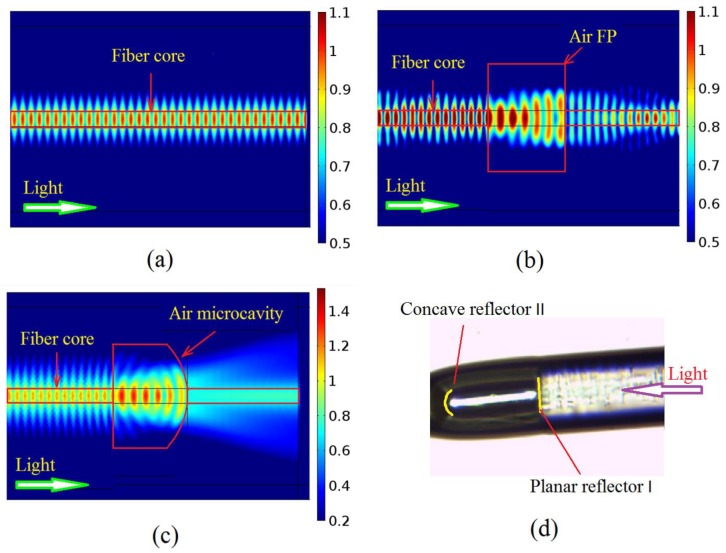
Mode field distribution base on FEM. (**a**) Mode field distribution of SMF. (**b**) Mode field distribution of F-P cavity. (**c**) Mode field distribution of F-P cavity with concave reflector. (**d**) Microscope image of F-P cavity with concave reflector. (SMF: Single mode fiber; the refractive index of the core is 1.4679; the refractive index of the cladding is 1.4613; the refractive index of air is 1; the incident light wavelength is 1550 nm.)

The process from Figure 1a,b is the optimization of spectral characteristics. The process from Figure 1b–d is the optimization of strain characteristics. This structure has better spectral characteristics than the traditional Fabry-Perot cavity: when the transmitted light is launched into the microcavity, the light will reflect on reflective surface I, forming Fresnel diffraction, as shown in Figure 2b, so this paper proposes that the reflective surface II be designed as a sphere with the best radius of curvature. It is known that a reflector II with extra-large or extra small curvature radius can also lead to scattering loss. If the reflected light energy is bound to the fiber core area, as shown in Figure 1d, an interference spectrum with higher power will be obtained. The spreading loss of reflection surface II is reduced by the focusing effect of concave mirror, as shown in Figure 2c.

**Figure 3:**
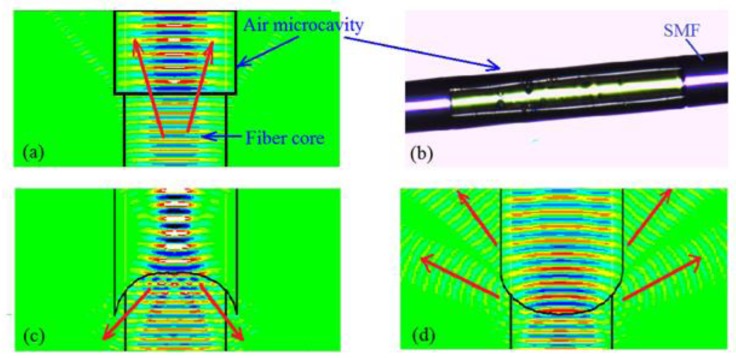
Analysis of the reflecting surface I based on FDTD. (**a**) Parallel reflecting surface I. (**b**) Microscope image of F-P with parallel reflector. (**c**) Concave reflecting surface I. (**d**) Convex reflecting surface I. (SMF: Single mode fiber; the refractive index of the core is 1.4679; the refractive index of the cladding is 1.4613; the refractive index of air is 1; incident light wavelength is 1550 nm; Light: 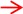.)

Why should the reflective surface I be perpendicular to the fiber axis? If the reflective surface is a curved surface that will lead to reflection loss, as shown in Figure 3c,d. Figure 3c will enhance reverse diffraction, and Figure 3d will enhance positive diffraction. Figure 3a is the most reasonable by comparison. Figure 3b is the physical figure of a parallel reflecting surface that indicates the feasibility and simplicity of preparation, but the curvature radius and cavity length of reflector II are difficult to control precisely, as shown in Figure 2d. We believe that higher precision preparation can be achieved with the development of the micro-/nano-3D printing [27]. In addition, the structure shown in Figure 1d has better strain sensitivity than the traditional F-P cavity: keeping the other structure parameters unchanged, the microcavity wall thickness and cavity diameter are reduced to improve the strain sensitivity. In practical applications, the hourglass optical microcavity strain sensor can be used in large bridges, ships and other micro-strain measurements [28].

### 2.2. Sensing Principle

The traditional Fabry-Perot interference principle is based on the theory of parallel plate multi-beam interference. The derived conditions of the theory [29] are: (1) consider the two reflective surfaces of the Fabry-Perot cavity are strictly parallel; (2) ignore the light spreading loss and the absorption loss of the reflective surface. However, the spherical reflector II of the hourglass optical microcavity structure is no longer strictly parallel to reflector I. The incident angle of light in reflective surface II has changed when it incides repeatedly on different positions of the reflective surface II. As is known from the Fresnel formula [19], the reflectivity of the same reflective surface is associated with the incidence angle of the light, so the multi-beam interference analysis of this structure is very complex. Research shows the interface reflectivity between optical fiber and air is less than 0.04, so we can use the double beam interference principle to simply analyze the hourglass optical microcavity sensor. The refractive index of air is 1. The interference light intensity of the sensor reflectance spectra is $I_{r}(\lambda )$ [29,30]:

(1) $$I_{r}(\lambda )=(R_{1}^{\prime }+R_{2}^{\prime }-2\sqrt{R_{1}^{\prime }R_{2}^{\prime }}\cos\frac{4\pi L}{\lambda })\cdot I_{0}(\lambda )$$

The maximum value of $I_{r}\left(\lambda \right)$ is $I_{\max}$:

(2) $$I_{\max}=(R_{1}^{\prime }+R_{2}^{\prime }+2\sqrt{R_{1}^{\prime }R_{2}^{\prime }})\cdot I_{0}(\lambda )$$

The interference contrast of the sensor reflectance spectrum is V:

(3) $$V=\frac{I_{\max}-I_{\min}}{I_{\max}+I_{\min}}=\frac{2\cdot \sqrt{R_{1}^{\prime }R_{2}^{\prime }}}{R_{1}^{\prime }+R_{2}^{\prime }}$$

In the formula, $R_{1}^{\prime }$ is an effective reflectivity of the reflective surface I; $R_{2}^{\prime }$ is an effective reflectivity of the reflective surface II, L is the microcavity length; λ is the wavelength of incident light; $I_{0}\left(\lambda \right)$ is the light intensity of the incident light; $I_{\max}$ is the maxima of interference spectral intensity; $I_{\min}$ is the minima of interference spectral intensity.

The strain sensing principle of the hourglass optical microcavity sensor is that the interference spectrum dip moves by the changing of microcavity length L. The deformation of the measured object makes the optical microcavity structure suffer an axial tension [31], and changes the microcavity cavity length, and this causes a reflection spectrum red shift. From Equation (1) the sensitivity of optical microcavity tension sensor can be obtained as follows:

(4) $$K=\frac{\partial \lambda_{dip}}{\partial F}=\lambda_{dip}\cdot \frac{\Delta L}{LF}$$

where *K* is the sensitivity of sensor; $\lambda_{dip}$ is the reflection spectrum peak/valley value; *F* is the axial tension; *L* is the microcavity length. From Equation (4), it is known that *F* is constant, and the sensitivity of the optical microcavity tension sensor mainly depends on ΔL/L.

## 3. Optimal Design of the Hourglass Microcavity Sensor

This section is divided into subheadings. It provides a concise and precise description of the experimental results, their interpretation as well as the experimental conclusions that can be drawn.

### 3.1. Influence of Microcavity Structure Parameters on Spectral Characteristics

**Figure 4:**
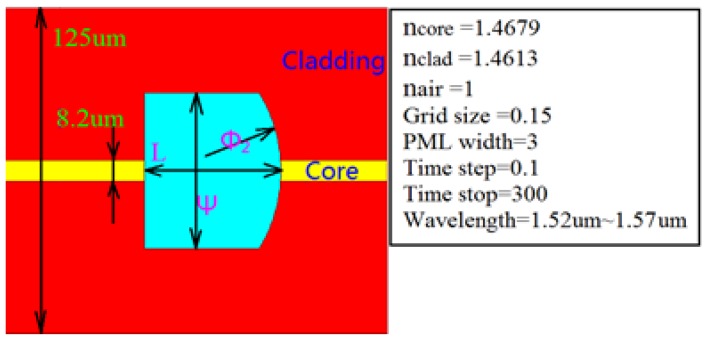
The simulation model of microcavity structure and parameter settings (the refractive index of the core is 1.4679; the refractive index of the cladding is 1.4613; the refractive index of air is 1; the wavelength range is 1520~1570 nm; the curvature radius of reflective surface II is $\phi_{2}$, microcavity wall is γ, microcavity diameter is ψ, microcavity cavity length is *L* ).

In this paper, FDTD is used to establish the microcavity structure model, and the interference spectrum signal intensity and contrast of the reflection spectrum are simulated, the optical microcavity structure parameters are mainly analyzed, including cavity length *L*, cavity diameter ψ and the curvature radius of the reflective surface II $\phi_{2}$. The parameters of the sensor by simulation are as shown in Figure 4.

**Figure 5:**
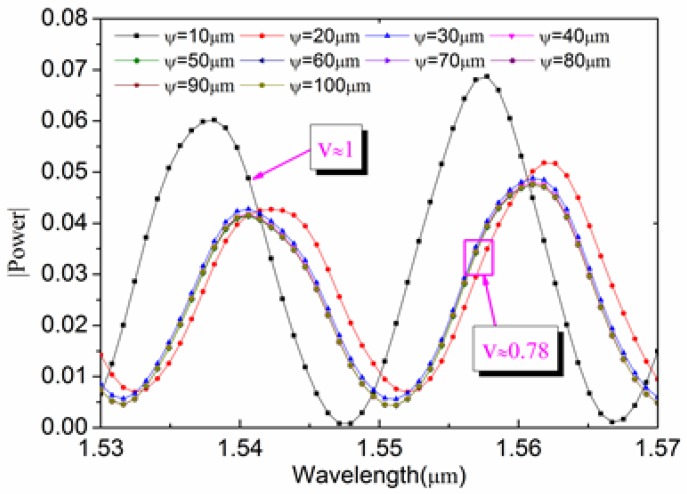
The microcavity interference spectra of different cavity diameters (wavelength range is 1520~1570 nm; the model parameters are: cavity length *L* of 60 μm, curvature radius of reflector II $\phi_{2}=\propto$, and cavity diameter ψ 10~100 μm.)

Before studying the influence of cavity diameter to contrast, a reasonable cavity length value $L_{0}$ and a curvature radius of reflector II $\phi_{2}$ must be given. From Equation (1), we can know that: $\begin{array}{cc} \frac{4\pi nL}{\lambda_{1}}-\frac{4\pi nL}{\lambda_{2}}=2k\pi & \left(k=0,1,2,3......\right) \end{array}$; n=1, $\lambda_{1}=1530$ nm, $\lambda_{1}=1570$ nm, when *k* is respectively 1/2/3, and the cavity length L is 30 μm/60 μm/90 μm, 1–3 dips respectively appear in the corresponding spectrum. Because the spherical reflector II of the optical microcavity will have a certain degree to influence the interference of reflected light, if the cavity length is 60 μm that can guarantee at least a dip in the spectral range of 1530 nm to 1570 nm, and it can avoid that mixing phenomenon because of the small free spectral range of the microcavity. Firstly, by selecting $\phi_{2}=\propto$, reflector II is plane. The interference spectrum simulation result is shown in Figure 5.

**Figure 6:**
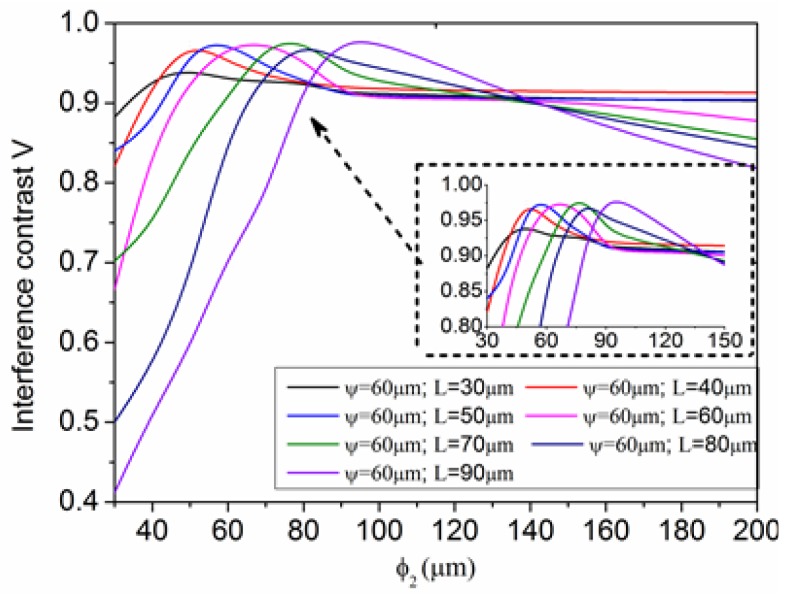
The curve of the microcavity interference spectrum contrast.

Figure 5 shows that the maximum value of the interference spectrum power (0.06~0.07) and the interference contrast (*V* ≈ 1) are all the best when ψ=10μm. The maximum value of interference spectrum power (0.04~0.05) and the interference contrast (*V* = 0.78) are almost unchanged when ψ = 2 ~10 μm. This indicates the cavity diameter's impact on the spectral characteristics. The reasons are as follows: when the microcavity diameter is less than the maximum width of the diffraction field, it constrains the diffraction of light, and part of the diffracted light will undergo reflection or total reflection on the microcavity wall, and at this time there will be more reflected light coupled back to the fiber core. It is worth noting that the cavity diameter can't be too small due to the difficulty of preparation, so reflector II's impact on the spectral characteristics is very important. The cavity length *L* and radius of curvature $\phi_{2}$ are related to the influence of the spectral characteristics based on the theoretical analysis. In this paper, the simulated cavity diameter is ψ=60 μm, the cavity length *L* is respectively 30~90 μm, and the curvature radius of $\phi_{2}$ is respectively 30~100 μm, 150 μm, 200 μm, ∝, and the interference spectrum contrast curve is as shown in Figure 6.

The abscissa in Figure 6 is the radius of curvature, the ordinate is interference contrast. Simulation found that the following parameters of microcavity have the best signal interference fringe contrast, and its signal intensity is greater than 0.08:

L=30 μm, $\phi_{2}=30\sim 50$ μm; L=40 μm, $\phi_{2}=40\sim 60$ μm; L=50 μm, $\phi_{2}=50\sim 70$ μm;

L=60 μm, $\phi_{2}=60\sim 80$ μm; L=70 μm, $\phi_{2}=70\sim 90$ μm; L=80 μm, $\phi_{2}=80\sim 90$ μm;

L=90 μm, $\phi_{2}=90\sim 110$ μm.

In order to fabricate the microcavity interferometer, a fusion splicer (Fitel, S178, Koga, Japan) and a mechanical fiber cleaver (S325 Fitel) are required in the experiment. The rectangular air FP cavity was made using a hollow-core fiber (HCF) section with a diameter of 50/125 µm sandwiched between two SMFs (SMF-28e+, Corning, NY, USA). The fusion parameter settings are as follows: discharge intensity of 110 unit; discharge time of 420 ms; first push distance of 8 µm, then stretch distance of 3 µm. After many experiments, we obtained a rectangular Fabry-Perot interferometer with a microcavity length of 60 μm. For the fabrication of the sensor with concave reflector, the spherical fiber end was made by electrical arc discharge on a section of HCF end face in a commercial fusion splicer.

**Figure 7:**
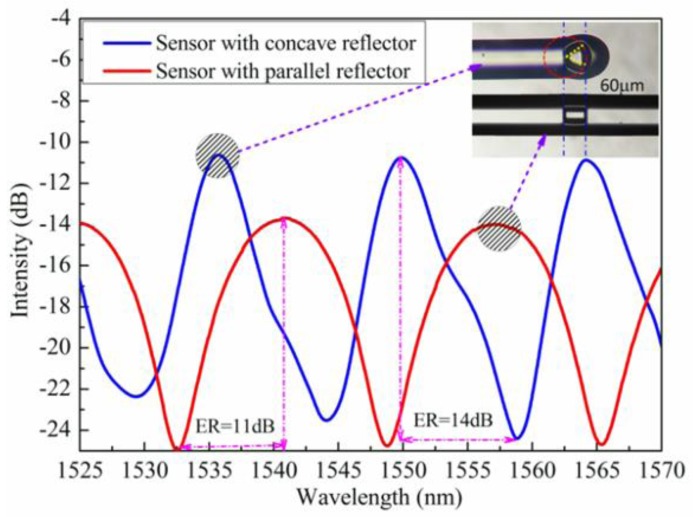
Experimental spectrum of the microcavity interference (blue line is the interference spectrum of the sensor with concave reflector; red line is the interference spectrum of the sensor with parallel reflector; ER: extinction ratio).

The experimental microcavity interference spectrum is shown in Figure 7. The extinction ratio is the difference between the peak value of the interference spectrum and the dip value of the interference spectrum. The experiment has been proved that the extinction ratio of Fabry-Perot interferometer with microcavity length 60 μm and concave reflector radius 60 μm is higher than the rectangular Fabry-Perot interferometer with microcavity length 60 μm (14 dB:11 dB). Due to the limitations of the experimental conditions, the experiment is not accurate enough, but it can match the simulation results.

### 3.2. Influence of Microcavity Structure Parameters on Strain Sensing Characteristics

**Figure 8:**
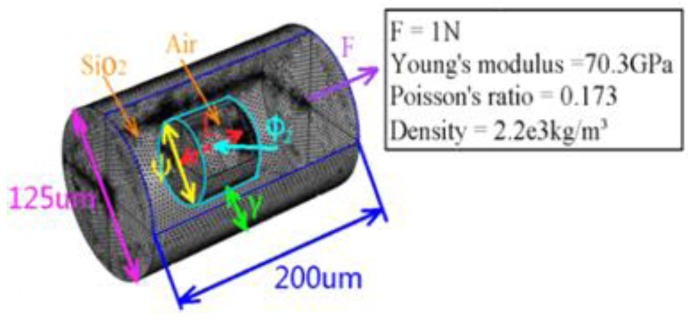
The simulation model of the hourglass microcavity structure and parameter settings.

From Equation (4), it is known that the sensitivity of an optical microcavity tension sensor mainly depends on ΔL/L when F is constant. This article uses the static mechanics of the finite element method to simulate the different size of hourglass microcavity structure, and gets the axial tension dependent variable. The parameters of the simulation model are shown in Figure 8, where the parameters settings are: the cavity diameter of the simulation model is ψ=60 μm, the cavity length *L* is respectively 30~90 μm and the curvature radius $\phi_{2}$ is 30~100 μm, 150 μm, 200 μm, ∝, while the other parameters remain constant.

**Figure 9:**
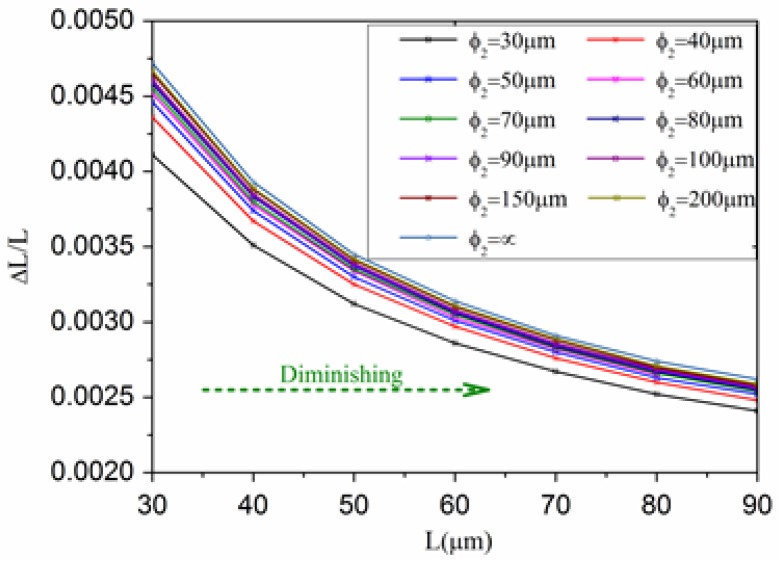
The relation curve between cavity length *L* and ΔL/L.

Figure 9 shows the relationship diagram between cavity length *L* and ΔL/L when $\phi_{2}$ changes. The abscissa is cavity length *L*, the ordinate is cavity length variable ΔL/L. The results show that when the optical microcavity cavity length *L* is the same,ΔL/Lincreases with the increase of curvature radius $\phi_{2}$. When the radius of curvature $\phi_{2}$ is the same, ΔL/L decreases with the increase of optical microcavity length *L*.

From mechanical knowledge, the thinner the cavity wall thickness, the easier the optical microcavity deformation [31]. Thus, this paper mainly studies the influence of cavity diameter ψ on cavity deformation with the same cavity wall thickness. Based on the analysis above, this paper chooses: cavity length L=40 μm, curvature radius $\phi_{2}=50$ μm, and the parameters of cavity wall and cavity diameter are as follows: ψ=60 μm, γ=32.5 μm; ψ=60 μm, γ=10 μm; ψ=40 μm, γ=10 μm.

**Figure 10:**
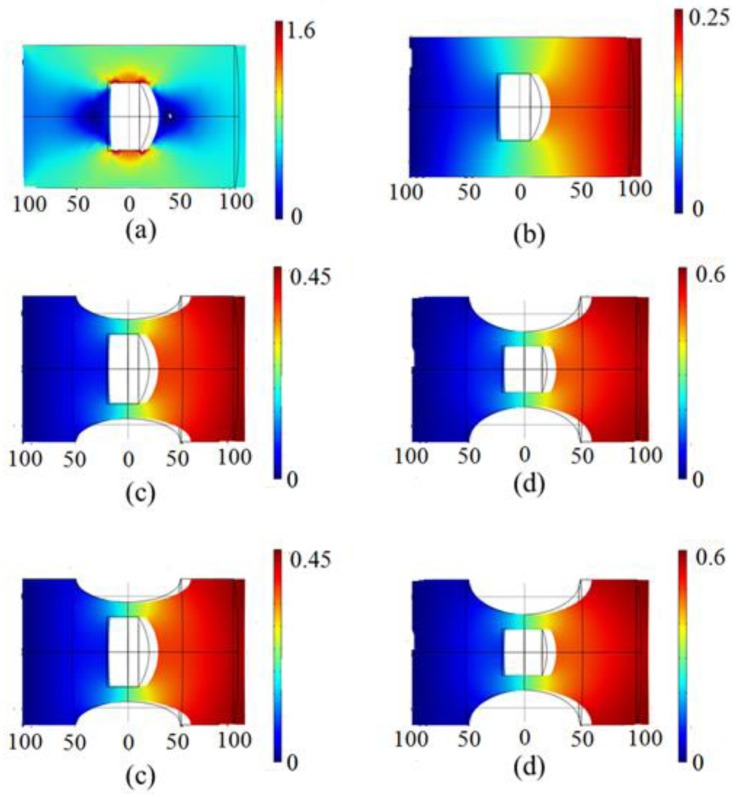
The simulation diagram of the microcavity model. (**a**) ψ=60 μm, γ=32.5 μm. The stress distribution of the stress distribution field (unit: N/m^2^); (**b**) ψ=60 μm, γ=32.5 μm. The displacement distribution field (unit: μm); (**c**) ψ=60 μm, γ=10 μm. The stress distribution of the stress distribution field (unit: N/m^2^); (**d**) ψ=40 μm, γ=10 μm. The displacement distribution field (unit: μm). (**e**) The relationship between ΔL/L and different values of cavity wall and cavity diameter.

Figure 10 shows the microcavity model field with different structural parameters under 1 N axial tension. Figure 10a is the stress distribution of the stress distribution field, Figure 10b–d are the displacement distribution fields. Blue color is minimum and the red color is the biggest in Figure 10. Figure 10a shows that when the optical microcavity sensor suffers axial tension stress, the cavity walls are the main stress area and two reflective surfaces are the minimum stress area. Therefore, we can ignore the influence of the curvature radius $\phi_{2}$ on the strain sensitivity. From Figure 10b,c, the microcavity structure deformation increases when the cavity diameter remains unchanged. Figure 10c,d showed that microcavity structure deformation increases along with the reduction of the cavity diameter when the microcavity wall thickness is unchanged. Figure 10e showed the increasing trend of ΔL/L along with the decrease of microcavity wall thickness and cavity diameter.

## 4. Sensing Properties of the Hourglass Optical Microcavity Sensor

**Figure 11:**
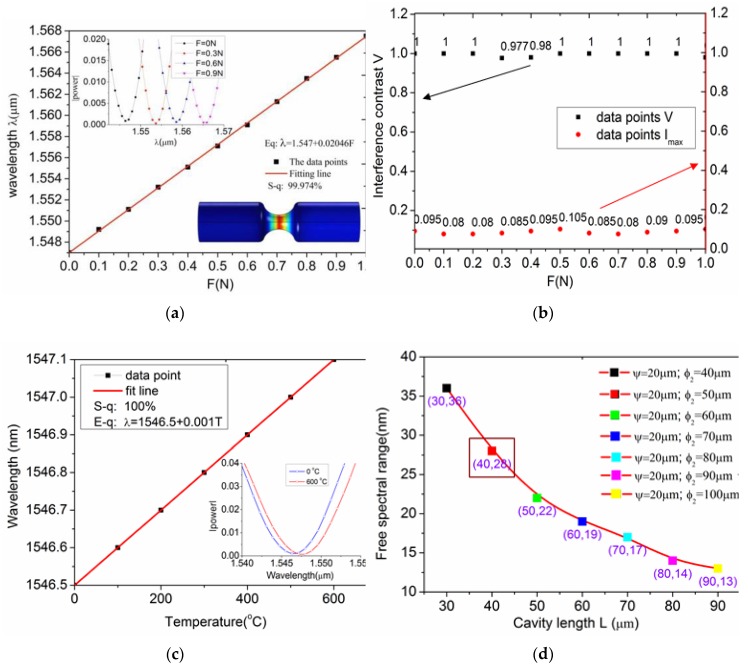
The sensing properties of the hourglass microcavity. (**a**) Strain sensitivity of the hourglass optical microcavity sensor. The inset shows the fringe dip shifts under different axial tension. (**b**) Interference contrast and light intensity reflectivity with different axial tension. (**c**) Temperature sensing properties. The inset is the wavelength shift with different temperature. (**d**) The free spectral range under different cavity length and curvature radius.

The sensor structure is put forward to further optimize the structure of the traditional Fabry-Perot interferometer, and has practical application value. The total length of interferometer structure given by simulation is 200 μm, microcavity parameters are ψ=20 μm, γ=10 μm, L=40 μm, $\phi_{2}=50$ μm. The axial tension range is 0–1 N that already can satisfy microstrain measurement demands in most cases according to the actual situation. The structure of the sensor is as shown in Figure 1d. The simulation results are as shown in Figure 11.

The inset in Figure 11a shows the wavelength shift to longer wavelength as the axial tension increases from 0–1 N (0–80 MPa). The fitting curve has a good axial-pull sensitivity of 20.46 nm/N and linearity of 99.947%. It is five times higher than the strain sensitivity of optical fiber strain sensor (about 4 nm/N) [32] in reports. The spectrometer resolution reaches to 1 mN based on the current equipment in laboratory.

Figure 11b shows that the interference intensity of reflection spectrum I is above 0.08 and the interference contrast of reflection spectrum is greater than 0.97, that is much better than the flat Fabry-Perot (*V* = 0.78, *I* = 0.04–0.05). That has important significance for small reflectivity sensors.

Figure 11c shows the temperature sensing properties of this sensor in the range of 0~600 °C based on finite element temperature field simulation. From the inset in Figure 11c it can be seen that a red-shift is observed with the increased temperature. It turns out that the temperature sensing properties of this sensor is only 0.001 nm/°C. Figure 11d shows the free spectral range of these structures:

L=30 μm, $\phi_{2}=40$ μm; L=40 μm, $\phi_{2}=50$ μm; L=50 μm, $\phi_{2}=60$ μm; L=60 μm, $\phi_{2}=70$ μm; L=70 μm, $\phi_{2}=80$ μm; L=80 μm, $\phi_{2}=90$ μm; L=90 μm, $\phi_{2}=100$ μm.

From the curve trend, we can see that the free spectral range decrease with the increase of curvature radius $\phi_{2}$. When the cavity length L=40 μm, ψ=20 μm and $\phi_{2}=50$ μm, the free spectral range is 28 nm that is very appropriate for 1520~1570 nm waveband.

## 5. Conclusions

An hourglass optical microcavity sensor structure is put forward in this paper. The sensor structure was analyzed based on theory and numerical simulation, and the theoretical calculation is consistent with the simulation results. The experimental results prove that the extinction ratio of an air microcavity Fabry-Perot interferometer with a cavity length of 60 μm and concave reflector radius of 60 μm is higher than that of a rectangular Fabry-Perot interferometer with a microcavity length of 60 μm. The hourglass microcavity strain sensor structure, compared with the common fiber Bragg grating strain sensor, has compact size, higher sensitivity, and temperature independence. The optimized structure obtained by simulation in this paper has an in-fiber microcavity length of 40 μm, microcavity wall of 10 μm, microcavity diameter of 20 μm and the curvature radius of reflective surface II is 50 μm. A good linearity of 99.947%, a resolution of 1 mN and a good axial-pull sensitivity 20.46 nm/N are achieved in the range of 0–1 N axial tension. The interference maximum intensity of the reflection spectrum is above 0.08. The intervening contrast of reflection spectrum is greater than 0.97. The results show that the proposed sensor structure has small volume, good mechanical strength, good quality spectrum, high sensitivity and so on.
